# Box embeddings for extending ontologies: a data-driven and interpretable approach

**DOI:** 10.1186/s13321-025-01086-1

**Published:** 2025-09-01

**Authors:** Adel Memariani, Martin Glauer, Simon Flügel, Fabian Neuhaus, Janna Hastings, Till Mossakowski

**Affiliations:** 1 Data Science Group (DICE), Heinz Nixdorf Institute, Paderborn University, Warburger Str. 100, 33098 Paderborn, North Rhine–Westphalia Germany; 2 Institute for computer science, Osnabrück University, Friedrich-Janssen-Str. 1, 49076 Osnabrück, Lower Saxony Germany; 3 Institute for Implementation Science in Health Care (IfIS), Faculty of Medicine, University of Zurich, Universitätsstrasse 84, 8006 Zürich, Switzerland; 4 School of Medicine, University of St. Gallen, (HSG), St. JakobStrasse 21, 9000 Gallen, Switzerland; 5 Institute for Intelligent Cooperating Systems, Otto von Guericke University, Universitätsplatz 2, 39106 Magdeburg, Saxony-Anhalt Germany

**Keywords:** Ontology, Box Embedding, Classification, ChEBI

## Abstract

**Abstract:**

Deriving symbolic knowledge from trained deep learning models is challenging due to the lack of transparency in such models. A promising approach to address this issue is to couple a semantic structure with the model outputs and thereby make the model interpretable. In prediction tasks such as multi-label classification, labels tend to form hierarchical relationships. Therefore, we propose enforcing a taxonomical structure on the model’s outputs throughout the training phase. In vector space, a taxonomy can be represented using axis-aligned hyper-rectangles, or boxes, which may overlap or nest within one another. The boundaries of a box determine the extent of a particular category. Thus, we used box-shaped embeddings of ontology classes to learn and transparently represent logical relationships that are only implicit in multi-label datasets. We assessed our model by measuring its ability to approximate the full set of inferred subclass relations in the ChEBI ontology, which is an important knowledge base in the field of life science. We demonstrate that our model captures implicit hierarchical relationships among labels, ensuring consistency with the underlying ontological conceptualization, while also achieving state-of-the-art performance in multi-label classification. Notably, this is accomplished without requiring an explicit taxonomy during the training process.

**Scientific contribution:**

Our proposed approach advances chemical classification by enabling interpretable outputs through a structured and geometrically expressive representation of molecules and their classes.

## Introduction

With the growing availability of chemical data in databases such as PubChem [1], there is a growing need to organise and make sense of the data. Classification of chemicals into classes based on structural features supports a number of data-driven applications, such as in drug discovery and systems biology [2]. Ontologies represent knowledge in a machine-interpretable way and support chemical classification in chemical ontologies such as ChEBI [3]. While ontologies have been successfully deployed in many domains, ontology development is a time-consuming and, thus, expensive effort [4]. For this reason, many ontologies only cover a fraction of the entities that are in their scope. For example, in the chemical domain, the ChEBI ontology currently includes only about 200,000 entities (of which only 61,000 are fully annotated), while the PubChem database contains about 120 million structurally distinct compounds, which are potentially in the scope of ChEBI. Thus, there is a demand for methods that can automatically extend existing high-quality ontologies, increasing their coverage of the domain. In order to ensure that the resulting ontology is coherent, it is important for this task that the extension follows the same classification principles and ontological architecture as the manually curated ontology.

In previous work, we showed that a transformer model was able to classify new chemical entities into the ChEBI ontology [5–7]. Despite its success, the previous work has two major limitations. Firstly, we use content from ChEBI to train models to classify chemicals, and – ideally – these trained models would learn to follow the same classification criteria that ChEBI developers apply. However, since the neural network architectures we used offer limited interpretability, it is difficult to evaluate whether the classification criteria our models learned are actually chemically correct. In [6, 7] we analysed the attention mechanism of a Transformer model in order to identify substructures of a given molecule that were relevant for its classification. Although this approach provides some indirect evidence to explain the classification, it does not enable us to directly evaluate whether the classification scheme the model learned is actually true.

Secondly, our previous work was limited to extending ChEBI with fully specified molecular structures (usually, the leaf nodes in the ChEBI hierarchy). Thus, we were not able to extend ChEBI with new classes of chemicals that include structurally diverse instances. E.g., our previous methodology enabled us to extend ChEBI with a class like *ethane*, since all ethane molecules share a common structure (i.e., $CH_3CH_3$), but not the class *alkane*, since two alkanes may have different molecular structures.

In this paper we present a new architecture that is intended to address both of these limitations: (1) The architecture enables more transparency in the sense that it is possible to inspect the taxonomy the model learned from the data and, thus, compare it with the ontology that it is intended to extend (i.e., ChEBI). And (2) the architecture enables the extension of the ontology with classes that represent structurally diverse members (like *alkane*). A core component of our methodology is the representation of classes as axis-aligned boxes within the model’s latent space. While learning to classify chemicals, the model learns the position and size of the boxes that represent chemical classes. The containment and non-overlap relationships between boxes in the latent space correspond to subsumption and disjointness relationships between classes. Thus, the spatial relationships among boxes in the latent space represent the taxonomy that has been learned by the model. This approach imposes strict constraints on how classes can be represented within the model’s embedding space. Despite these constraints, the performance of the proposed model in classifying previously unseen chemical structures matches that of the best models from our earlier studies, as outlined in [5–7].

*Related Work.* The functionality of classifying chemical structures is also provided by Classyfire [8], which follows a rule-based approach and, thus, requires manual maintenance to extend its coverage (see discussion in Sect. 4.1). While our approach is inspired by previous work on box embeddings [9, 10], our goals are different and, thus, the direction is reversed: In those previous works, the authors embed a given ontology in a latent space as boxes based on the logical axioms of the ontology. In contrast, we train a model to classify chemicals in a way that we can inspect the taxonomic relationships between the representations of the classes it learned. Afterward, we evaluate the quality of the model by comparing these relationships with the logical axioms in ChEBI.

In the following Methods section, we present our approach in detail. In the subsequent Evaluation section, we first evaluate the performance of our approach, and compare our results with previous models on the same tasks and datasets. We then assess how effectively our approach handles the newly introduced tasks of hierarchy and disjointness inference. In the Discussion section, we explore the implications and limitations of our approach and compare it to related work. Finally, we conclude with an outlook on future work.

## Methods

Here, we present our training dataset, model architecture, and the application of the proposed method for ontology extension.

### General approach

We approach the problem of ontology extension as a hierarchical multi-label learning task. Therefore, we use a dataset for the classification of chemicals, which has already been successfully used in our previous works across a range of learning methods [6, 11], and is based on the ChEBI ontology (see Sect. 2.2 for more details). The training goal is to correctly assign ChEBI classes to the molecules based on their SMILES representations [12].

The ChEBI ontology organizes chemical entities into a hierarchical taxonomic structure. As an example, according to this hierarchy, *acetic acid*(CHEBI:15366) is a subclass of *carboxylic acid*(CHEBI:33575), which, in turn, is a subclass of *organic acid*(CHEBI:64709).

From an ontological perspective, ChEBI is composed of chemical classes. However, to formulate a machine learning classification task, we treat these classes based on structural similarity. Therefore, chemical classes composed of structurally identical compounds, and representable by a single SMILES string, are treated as instances in our dataset. In addition, classes that contain structurally diverse instances are considered as labels. For example, *acetic acid* is treated as an instance, and both *carboxylic acid* and *organic acid* are considered as labels. Since the labels are based on classes in ChEBI, they inherit the hierarchical structure from the ontology.

Thus, if, for example, a model classifies a given chemical compound as *carboxylic acid*, it should also classify it as *organic acid*.

If a model fails to do that, it is inconsistent with the subsumption relationship between the classes, which shows that the model failed to learn an accurate internal representation of chemical reality. Unfortunately, the opposite does not hold true: If a model classifies a dataset such that any chemical structure that is labeled *carboxylic acid* is also labelled as *organic acid*, this nevertheless does not guarantee that the model learned the chemical relationship between *carboxylic acid* and *organic acid* accurately. Because it could be an accident that the dataset does not contain an example of a molecule for which a predicted label is inconsistent with the subsumption relationship.

One major goal of our approach is to address this issue by introducing a novel architecture for models that (a) are able to extend ChEBI by automatically classifying chemical structures in a way that (b) it is possible to evaluate whether a trained model is chemically accurate by inspecting the taxonomical relationships it learned. For this purpose, we propose an approach that couples the classification task with learning a class hierarchy. By comparing the taxonomy that has been learned by the model with the ontology, specifically ChEBI, we can validate whether the model actually learned the semantic relationships in the ontology accurately. Further, this approach enables us to extend the ontology with classes that contain structurally diverse instances.

To achieve this goal, we formulated a new learning problem to construct a taxonomy of labels represented as boxes, with data instances embedded as points in the same space. This learning problem can be tackled using two primary methodologies: (1) A *data-driven* approach where relationships between labels are inferred directly from the data instances. Here, it is worth mentioning that a data instance can have several labels and therefore should be placed inside multiple boxes in the embedding space. (2) A *semi-supervised* approach to incorporate domain-specific constraints into the training process, typically through the design of a custom loss function. These constraints leverage expert knowledge or pre-existing ontologies to guide the learning process and ensure alignment with previously established domain rules. For example, subsumption and disjointness axioms from an existing ontology can be integrated into the learning process as constraints that must be respected during the training phase. As practical implementations in this direction, earlier entity typing models have integrated explicit hierarchies using hierarchical loss functions to capture the relationships between entity types [13, 14]. However, these approaches rely on an explicitly defined hierarchy.

In this paper, we focus exclusively on the data-driven approach, which addresses multi-label learning problems without relying on any pre-defined taxonomical structures for label associations. The labels in the dataset are used to train the model to represent classes as boxes in the embedding space. Since the spatial relationships between these boxes are representations of subsumption and disjointness relations between the corresponding classes, the trained model corresponds to a new ontology, which may be expressed, e.g., as an OWL file and be compared to ChEBI. The exploration of the second methodology, involving the integration of domain-specific constraints, is planned for future work.

### Dataset

In the ChEBI ontology, chemical structures are represented as classes. Some of these classes are very general (e.g., *molecular entity* (CHEBI:23367)) while others express specific features that are shared by a variety of different chemical structures (e.g., *aromatic alcohol* (CHEBI:33854)), and some classes represent chemicals that have a single structure. For example, all instances of the class *benzyl alcohol* (CHEBI:17987) share the same molecular structure, which is represented in ChEBI by the SMILES string OCc1ccccc1. While ChEBI represents *benzyl alcohol* and *aromatic alcohol* in the same way, in the dataset introduced in [6, 11], they are treated differently: *benzyl alcohol* as an instance, and *aromatic alcohol* as a class. More precisely, a class like *benzyl alcohol*, whose members are structurally identical and can be represented by a single SMILES string, is treated as a data point.

**Fig. 1:**
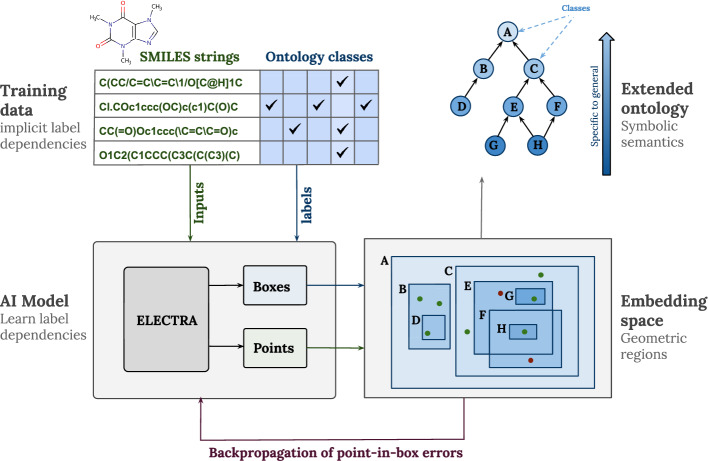
Label dependencies are implicit within our training database, and our model makes them explicit as *learned* boxes in the embedding space. In addition, SMILES strings are embedded as points

The other classes are labels of these datapoints (see Fig. 1, top left), which are used for a classification task. In other words, for the purpose of training our models we treat *benzyl alcohol* as a member of classes that are represented by labels in the dataset (e.g., *aromatic alcohol*, *alcohol*, *molecular entity* etc.). Hence, class assertions (for instances) in the context of this paper correspond to subsumption relationships in ChEBI.

While in OWL2 DL, negative class assertions can be expressed, negative subsumption relations cannot. Accordingly, we do not have negative information in our dataset, and take a closed-world view, i.e. subsumption relations that are not specified (and cannot be inferred either) are considered not to hold.

### Box embedding architecture

In our previous work [6, 11, 15], we used SMILES annotations to develop a classification system that can predict which superclasses a class would have in ChEBI based on a SMILES string representing the structure of that class. These systems, however, had two downsides. First, it was unknown whether these systems learned the underlying hierarchy of the data and, second, the approach was limited to chemical classes associated with a SMILES string. Further, extending these models to include new classes required retraining, which is time and energy-intensive. We aim to overcome these downsides by employing a geometric representation. Our proposed approach is applicable to any ontology with structured annotations.

As depicted in Fig. 1, our model consists of two components. The first is a representation of boxes as trainable parameters. Given a class *C*, its box – denoted as ${{\mathcal {B}}_{C}} = ({{\mathcal {B}}_{C}}^1, {{\mathcal {B}}_{C}}^2)$ – is represented as a pair of two *n*-dimensional corner points ${{\mathcal {B}}_{C}}^1, {{\mathcal {B}}_{C}}^2 \in {\mathbb {R}}^n$. Second, we use an Electra-based [16] model that has already been successfully employed for prediction tasks on the given dataset [6, 7, 11]. Each SMILES string is tokenized and transformed into a sequence of chemically meaningful tokens, as done in [6, 7]. Our proposed model uses an additional projection head (a single fully connected layer) that maps Electra’s 256-dimensional outputs to *n*-dimensional vectors (points). In our model, classes are represented as axis-aligned boxes in the same n-dimensional space as points, and training optimizes both the points and the box parameters using a point-in-box containment objective. This yields a shared (“co-embedding”) space for molecules and classes. Consequently, the final embedding dimension is n-dimensional (not 256); we found n = 16 to perform best. In summary, the model processes a given SMILES string and embeds it as a point $p \in {\mathbb {R}}^n$, while jointly optimizing the representation of the boxes for ChEBI classes.

As illustrated in Fig. 1, if a class *A* subsumes class *B* (i.e., *A* is more general than *B*), then in the embedding space, the box that represents class *A* should *contain* the box that represents class *B*. Analogously, a SMILES embedding contained within a box can be interpreted as a subclass assertion for the corresponding ontology class. Because boxes are closed under intersection (i.e., if some boxes overlap, their intersections remain a box), geometric box intersection can be interpreted as logical *conjunction*. Thus, if a class X coincides with the intersection of two boxes Y and Z, it may be interpreted that X is equivalent to the conjunction of Y and Z (i.e., as the axiom $X \equiv Y \sqcap Z$ in description logic notation).

Box embeddings can also model class disjointness by representing two disjoint classes as disjoint boxes in the embedding space. For example, classes B and C in Fig. 1 do not overlap.

As discussed above, one motivation for the approach illustrated in Fig. 1 is to enhance the existing work on ontology extension. Yet, the approach does not depend on a pre-existing ontology, because the training only uses the labels in the training data (in our case, SMILES strings). Using the data, our proposed system will learn hierarchical relationships among the labels and thus create a new ontology that incorporates these labels as ontological classes. This may be particularly interesting for domains lacking a well-established reference ontology. If ontology extension became our sole focus, the approach in Fig. 1 could be extended, for example, by modifying the loss function to account for unsatisfied axioms from the ontology. However, since we intend to evaluate our approach for both use cases (i.e., extending an existing ontology and learning a new ontology from data), in the sequel, we do not use ChEBI’s axioms during the learning process, but only for evaluation purposes.

### Box membership

A molecule represented by a SMILES string *x* is considered a member of a class *C* if the embedding *p*(*x*) of the molecule is inside of the box ${{\mathcal {B}}_{C}}$. The goal during training is therefore to minimize the distance of the embedding *p*(*x*) to all boxes of chemical classes containing *x*. We can define the distance of *p* to the box ${{\mathcal {B}}_{C}}$ as

$$\begin{aligned} d_i(p, {{\mathcal {B}}_{C}}):= \max (0,(l^{{\mathcal {B}}_{C}}_i + {\varepsilon _{\text {in}}}\cdot v^{{\mathcal {B}}_{C}}_i) - p_i, p_i - (r^{{\mathcal {B}}_{C}}_i - {\varepsilon _{\text {in}}}\cdot v^{{\mathcal {B}}_{C}}_i)). \end{aligned}$$

Here $l^{{\mathcal {B}}_{C}}:={\min }({{{\mathcal {B}}_{C}}}^1, {{{\mathcal {B}}_{C}}}^2)$ and $r^{{\mathcal {B}}_{C}}:={\max }({{{\mathcal {B}}_{C}}}^1, {{{\mathcal {B}}_{C}}}^2)$ are the element-wise minimal and maximal corners of the box representing class *C*. Consequently, $l^{{\mathcal {B}}_{C}}_i$ and $r^{{\mathcal {B}}_{C}}_i$ are the left and right corners in the projection of this box on dimension *i*. The dimension-wise width of the box $v^{{\mathcal {B}}_{C}}:= r^{{\mathcal {B}}_{C}} - l^{{\mathcal {B}}_{C}}$ and the hyperparameter ${\varepsilon _{\text {in}}}$ dictate the size-dependent ‘safety margin‘ that shrinks the box slightly for numerical stability. Note that this distance is 0 for all dimensions in which the given point lies between the faces of the box. We can then define the membership of a given point in a box as:

$$\begin{aligned} m(p, {{\mathcal {B}}_{C}}):= 2 \cdot \left( 1 - \sigma \left( \sum \limits _{i=1}^{n} d_i(p, {{\mathcal {B}}_{C}}) \right) \right) \end{aligned}$$

**Fig. 2:**
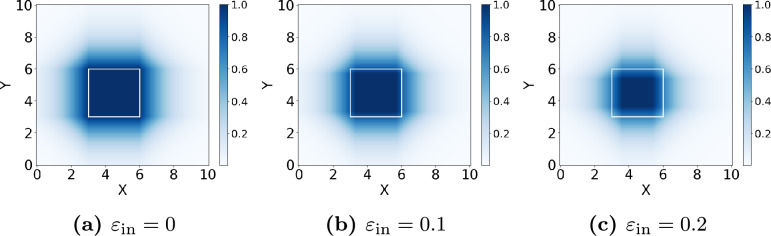
Membership function for different margins

The sigmoid function $\sigma$ facilitates a smooth transition between membership degrees, yielding a value of 1 inside the box and gradually approaching 0 as the distance from the box increases. Figure 2 shows the membership function for different margins. We use margins dependent on the size of boxes to allow the model to learn boxes that are smaller than any fixed margin. We train our model on a dataset that has been derived from the ChEBI ontology and that has already been used for similar ontology-based learning tasks [6, 11]. This dataset consists of all SMILES strings that are attached to a subclass of *molecular entity* (CHEBI:23367). Likewise, the target labels are all 854 classes in ChEBI that have at least 100 subclasses that are annotated with a SMILES string.

### Loss function

The loss of our model is calculated based on a weighted binary cross-entropy where *p*(*x*) is the embedded point in the latent space for a given SMILES string *x* and $w_C$ is a class-specific weight.

1 $$\begin{aligned} L(x, y) = \sum _{C \in {\mathcal {C}}} w_C \cdot y_C \cdot \log m(p(x),{{\mathcal {B}}_{C}}) + (1 - y_c) \cdot \log (1 - m(p(x),{{\mathcal {B}}_{C}})) \end{aligned}$$

Due to the hierarchical structure of the ChEBI ontology, the training dataset is inherently imbalanced; therefore, applying weights in the loss function is critical to ensure effective learning. It has been observed in earlier research that an additional weighting scheme improves the predictive performance [11]. We also adopted this strategy in our work.

2 $$\begin{aligned} w^{\text {Cui}}_c = \frac{1 - \beta }{1 - \beta ^{|c|}} \quad \quad w_{c}^{norm} = \frac{N_{s}}{\left| C \right| \cdot N_{l}} \end{aligned}$$

The weighting $w^{\text {Cui}}_c$ was originally introduced by Cui et al. [17] and is based on the class size |*C*|. During training, we used the parameter $\beta =0.99$ as recommended by Cui et al.

However, this weighting scheme applies only a marginal scaling to classes; the ontology-based multi-label dataset in this study features some classes that contain all datapoints, while others contain only 100. Therefore, we also experimented with a second weighting scheme $w_{c}^{norm}$ to prioritise minority classes by assigning them significantly higher weights.

In definition 2, $N_{s}$ is the total number of samples, $N_{l}$ is the number of labels, and $\left| C \right|$ is the number of samples in class C. Due to the ontological origin of the dataset, minority classes are often more specific classes.

We trained the model on a single GPU for 600 epochs. We empirically observed that our best results were achieved with 16 dimensions, as performance deteriorates in higher dimensions. In Sect. 4, we will discuss more about the effect of dimensionality on the learning process.

### Inference

The training system described above yields a method to train a *box- and point-based prediction* model for ontology-based classification. However, the primary focus of our study was the question of whether the resulting box representations for classes are consistent with the subsumption hierarchy of the ontology. To this end, we infer that a class *A* subsumes a class *B* iff the box ${{\mathcal {B}}_{B}}$ is contained in a box ${{\mathcal {B}}_{A}}$ up to a certain margin. More precisely, that means we infer that $B \sqsubseteq A$ iff in every dimension $i \in \{1,\ldots ,n\}$ it holds that $l^{{\mathcal {B}}_{A}}_i \le l^{{\mathcal {B}}_{B}}_i + {\varepsilon _{\text {sub}}}\cdot v_i^{{\mathcal {B}}_{B}}$ and $r^{{\mathcal {B}}_{B}}_i - {\varepsilon _{\text {sub}}}\cdot v_i^{{\mathcal {B}}_{B}} \le r^{{\mathcal {B}}_{A}}_i$. The additional ${\varepsilon _{\text {sub}}}$ hyperparameter is necessary because initial tests have shown that the edges of boxes are subject to subtle wobbling during training. This is due to the different ways in which the training gradient influences boxes of different sizes. The direct edges of the boxes are therefore often imprecise. Just as we used a margin term in the definition for the loss function to counteract this behaviour, we use the hyperparameter ${\varepsilon _{\text {sub}}}$ in the detection of the subsumptions.

Similarly, we derive a disjointness relation between two classes *A* and *B*. We also employ a scaling factor to counter the instabilities introduced by the training algorithm. The scaling factor is applied to the sum of both box sizes instead of one of the boxes because the disjointness relation is symmetric. Therefore, we consider two classes disjoint, i.e. $B \sqsubseteq \lnot A$, iff for some $i \in \{1,\ldots ,n\}$ it holds that $r^{{\mathcal {B}}_{B}}_i - {\varepsilon _{\text {dis}}}\cdot (v^{{\mathcal {B}}_{A}}_i + v^{{\mathcal {B}}_{B}}_i) < l^{{\mathcal {B}}_{A}}_i$ or $r^{{\mathcal {B}}_{A}}_i - {\varepsilon _{\text {dis}}}\cdot (v^{{\mathcal {B}}_{A}}_i + v^{{\mathcal {B}}_{B}}_i) < l^{{\mathcal {B}}_{B}}_i$, i.e., classes with minimally overlapping boxes are still considered to be disjoint. As the opposite of disjointness, we also use an overlap relation, i.e., $B \not \sqsubseteq \lnot A$. This relation holds iff for all $i \in \{1,\ldots , n\}$ $r^{{\mathcal {B}}_{B}}_i - {\varepsilon _{\text {dis}}}\cdot (v^{{\mathcal {B}}_{A}}_i + v^{{\mathcal {B}}_{B}}_i) \ge l^{{\mathcal {B}}_{A}}_i$ and $r^{{\mathcal {B}}_{A}}_i - {\varepsilon _{\text {dis}}}\cdot (v^{{\mathcal {B}}_{A}}_i + v^{{\mathcal {B}}_{B}}_i) \ge l^{{\mathcal {B}}_{B}}_i$. This yields prediction methods for subsumption, disjointness, and overlap relations between classes. We will evaluate these relations with the respective axioms as represented in ChEBI. We also analyse whether these predictions satisfy the logical theory of the ontology. Given a subsumption relation $A \sqsubseteq B$ or a disjoint relation $A \sqcap B \sqsubseteq \bot$, we calculate the number of violations that are produced by a trained system.

### Extension with unseen classes

The system defined in this way classifies instances in classes based on the spatial position of the instance points in the embedding space within class boxes. However, it is an essential part of ontology development that new classes are added to an ontology, not only that instances are classified in existing classes. Accordingly, the question arises as to whether this representation can also be used to add new classes to an existing ontology and annotate them with corresponding subclass axioms. A potential use case would be a given new class *C* for which a given set of members is already known, e.g., a new class of chemicals with a set of molecules that instantiate that class.

Our box-based approach can also be used to dynamically integrate such new classes by deriving new boxes without retraining. For a new class *A* we create a new bounding box that contains all instances $a_i, \cdots a_n$ that represent members from *A*:

$$\begin{aligned} l_i^{{\mathcal {B}}_{A}} = \min _{j=1,\cdots , n}(a^j_i), \qquad r_i^{{\mathcal {B}}_{B}} = \max _{j=1,\cdots ,n}(a^j_i) \end{aligned}$$

To analyse this feature of our model, we also tested the behaviour of the model on previously unseen classes for zero-shot subsumption prediction. The dataset used for training was restricted to classes that contain at least 100 members in ChEBI. For the zero-shot learning analysis, we consider all classes in ChEBI that have at least two SMILES-annotated subclasses and that have not been used as training labels. Since our dataset consists of all SMILES strings contained in ChEBI, these 9,805 additional classes have at least two members in our dataset.

We then compare the emerging new subsumption relation to other boxes according to our earlier definition and compare them to those defined in ChEBI. Notably, this approach is particularly challenging because classes that reside further down in an ontological taxonomy have significantly lower evidence than those further up.^1^

## Evaluation

To evaluate the general prediction quality of our system, we compare the predictions of the system with the ground truth from the ChEBI ontology. For this purpose, we consider three different measures: precision, recall, and the harmonic mean of these values, the F1 score.

As already mentioned, the training dataset is highly imbalanced due to its ontology-based origin. Therefore, not all common measures of predictive quality are suitable for evaluations on this dataset. In the literature, the measures mentioned above are often calculated globally. However, this method - also known as *micro-aggregation* - weights all predictions equally. As a result, in the case of unevenly distributed classes, it favours those that occur particularly frequently. In ontology-based classification, however, this means that those classes that are particularly high up in the taxonomy are focused on. However, these classes are often very general and not particularly interesting for the classification of chemicals. We therefore use two further quality measures that allow a better insight into the application-oriented quality of the prediction model. A *macro* metric is obtained by calculating the corresponding metric for each class individually and then averaging them. This means that all classes are weighted equally, which in turn strongly favours classes that have particularly few instances.

Consider the classes aldehyde (CHEBI:17478) and the corresponding subclass aliphatic aldehyde (CHEBI:59768). If the model examines a molecule that actually belongs to the class aliphatic aldehyde, but classifies it only as an aldehyde, this is not incorrect, just logically imprecise. An equal weighting of all classes, therefore, places too strong a focus on very specialised classes. Therefore, in addition to the micro- and macro-aggregated measures, we also calculate a weighted variant of the macro-metrics. This metric combines the class-based aggregation of the macro-F1 score, but puts a higher emphasis on more frequent targets, by weighting them with the total number of instances in the respective class [18].

**Table 1:** F1 scores for the molecule classification task

	Micro			Weighted			Macro		
	Precision	Recall	F1	Precision	Recall	F1	Precision	Recall	F1
$w^{\text {Cui}}$	**0.930**	0.885	**0.907**	**0.919**	0.885	**0.897**	0.726	0.525	0.580
$w^{\text {norm}}$	0.901	0.813	0.854	0.893	0.813	0.845	**0.826**	**0.620**	**0.687**
Electra	0.915	**0.887**	0.901	0.903	**0.887**	0.890	0.664	0.511	0.540

Table 1 shows the evaluation metrics for the ontology-based classification task. The baseline model is an Electra model without the additional geometric embeddings (*Boxes*). It can be seen that the model that uses the $w^{\text {Cui}}$-weighting during training performs better regarding the micro and weighted aggregation than the one using the $w^{\text {norm}}$ weighting, and also slightly better than the baseline model. It is, however, outperformed by the model using the $w^{\text {norm}}$ weighting on the macro aggregation. This behaviour is attributed to the fact that the $w^{\text {norm}}$ metric strongly de-emphasizes classes that have a large number of members. This decreases the quality of the average per-molecule predictive power by biasing the model towards learning rarer, more specific classes (which are also more interesting for the chemist). The F1 scores of the model using the $w^{\text {Cui}}$ match those of models trained without the additional representation of classes as boxes. The predictive performance has therefore not deteriorated significantly. It should, however, be noted that even though it has worse predictive performance overall, the model trained with $w^{\text {norm}}$ weighting shows better predictive performance for classes that reside lower in ChEBI’s taxonomy. The comparison to the baseline model indicates that the additional n-dimensional geometric representation for the boxes does not have a negative impact on the predictive performance.

### Subsumption

**Fig. 3:**
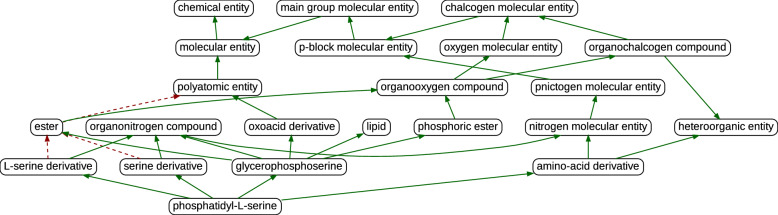
Class hierarchy derived from box containment. Using an arbitrary class from the dataset such as phosphatidyl-L-serine, we computed its parent classes through geometric containment. Green arrows show box containment relations that correspond to the *is_a* relations in ChEBI, red dashed arrows show box containment relations not found in ChEBI

The second part of our analysis concerns the hierarchical relationships between classes that are induced by the containment of their respective boxes. Our proposed approach allows us to derive a hierarchy from the boxes that represent ChEBI classes in the embedding space. Figure 3 shows an example of the derived hierarchy for a given class. The green arrows show box containment relations that correspond to the *is_a* relations in ChEBI’s inferred class hierarchy. The red arrows show those not found ChEBI. In the box-derived hierarchy shown in Fig. 3, *L-serine derivative* appears to be a subclass of *ester*. While this may hold for certain specific derivative of L-serine, ChEBI does not classify *L-serine derivative* as a subclass of *ester* because this relationship does not hold as a general rule. Nevertheless, in some cases, the box model can be used to suggest possible "could be" relations that may help enrich the ontology. For example, in ChEBI, a relation between *ester* and *polyatomic entity* is missing. However, the box-derived hierarchy produced this relation, which, while absent from ChEBI, is in fact correct.

**Fig. 4:**
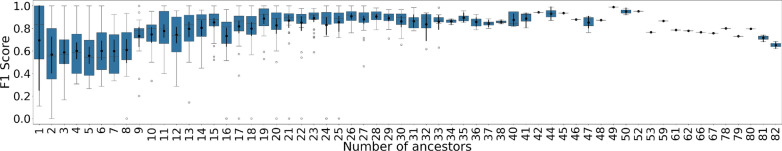
$w^{\text {Cui}}$ grouped F1 scores by number of ancestors

**Fig. 5:**
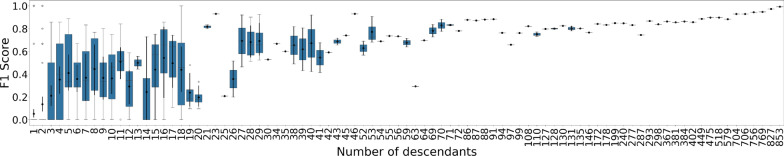
$w^{\text {Cui}}$ grouped F1 scores by number of descendants

**Fig. 6:**
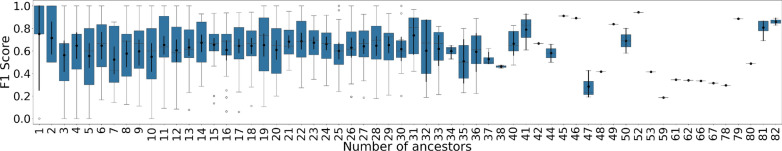
$w^{\text {norm}}$ grouped F1 scores by number of ancestors

**Fig. 7:**
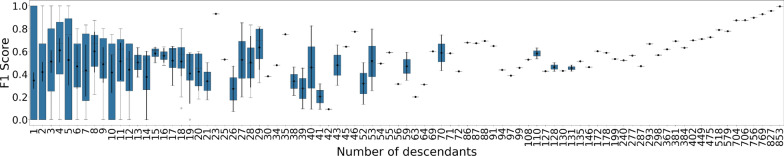
$w^{\text {norm}}$ grouped F1 scores by number of descendants

**Fig. 8:**
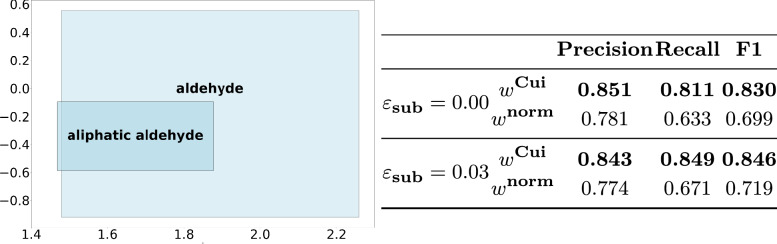
Almost-containment between trained boxes

In ChEBI, classes further down in the hierarchy often tend to have fewer subclasses. Figures 4, 5, 6 and 7 show the grouped F1 scores for both models in inferring the hierarchy among classes. The scores are categorized based on the number of ancestors (superclasses) and descendants (subclasses). These results show that the $w^{\text {norm}}$ model was more effective in inferring a better hierarchy for rare and more specific classes. A comparison of Figs. 5 and 7 reveals that Fig. 7 shows better F1 scores for more specific classes—those with very few descendants in the ChEBI hierarchy. Furthermore, while calculating the geometric box containments to infer subsumption relations, we noticed that the spatial properties of some boxes representing specific classes do not perfectly adhere to the expected hierarchy. Therefore, we examined how these boxes were shaped and observed a common pattern in the unfavourable parts of the inferred hierarchy: the misplaced boxes are mostly contained in the expected boxes, with only a small part remaining outside. Figure 8 shows an example of an overlooked subsumption relation in our evaluation: the 16-dimensional learned boxes for *aldehyde*(CHEBI:17478) and *aliphatic aldehyde*(CHEBI:59768) are projected into a 2-dimensional space. As it is shown, *aliphatic aldehyde* as the child class is mostly contained in its parent class *aldehyde*. However, the small part that lies outside of the parent class led to a misinterpretation regarding the expected hierarchical relationships between the two classes. In our evaluations, we view boxes as *rigid* n-dimensional overlapping intervals. As stated in [19] and [20], the *‘hard edges’* of boxes make gradient-based optimization challenging. Therefore, we hypothesized that box misplacements often have roots in numerical issues. To address the issues regarding the *hard* interpretation of box boundaries, we introduced a small allowed margin ${\varepsilon _{\text {sub}}}$ for numerical errors in our evaluation, and it led to a significant improvement regarding the learned subclass relations. The results of this modification are presented in the remaining tables and figures, where we have included the $\varepsilon$ in our evaluations. The allowed margin of error for each dimension is determined by the length of the containing box in that dimension multiplied by the epsilon value. For example, an ${\varepsilon _{\text {sub}}}$ of 0.03 reduces the size of the contained box by 3%.

Table 2 shows the results for the micro F1 score of the induced hierarchy in comparison with the hierarchy contained in the transitive closure of the ChEBI. Similar to the results reported for the overall predictive quality, the ${w^{\text {Cui}}}$ weighting generally outperforms the model trained with ${w^{\text {norm}}}$ weighting when considering the total number of true positives, false positives, and false negatives class relationships. While in usual classification tasks, these metrics are used to evaluate a binary relation between instances and classes, the subsumption relationships we intend to evaluate here concern the association between classes. There are, therefore, different ways to calculate the usual class-based aggregations. Here, for hierarchy and micro-aggregation, subsumption counts remain the same. However, in macro and weighted aggregations, the counts vary depending on whether subclasses or superclasses are considered. For a given class, we collect the inferred subclasses and the inferred superclasses and analyze them separately. Table 3 shows the results of these individual assessments. While the model trained with the $w^{\text {Cui}}$ weighting outperforms in all metrics for the predicted superclasses, it is outperformed when considering the macro aggregation for the subclass relationships.

**Table 2:** Micro-aggregated evaluation for derived subsumption relations

		Precision	Recall	F1
${\varepsilon _{\text {sub}}}= 0.00$	$w^{\text {Cui}}$	**0.851**	**0.811**	**0.830**
	$w^{\text {norm}}$	0.781	0.633	0.699
${\varepsilon _{\text {sub}}}= 0.03$	$w^{\text {Cui}}$	**0.843**	**0.849**	**0.846**
	$w^{\text {norm}}$	0.774	0.671	0.719

These results are consistent with our results on predictive performance, which we have already reported. Classes in the ChEBI taxonomy have fewer subclasses than any of their parent classes. Further, specific classes generally have fewer subclasses than more generic classes. At the same time, these classes also have an inferior prediction quality in the classification task, which implies that the spatial properties of the learned boxes (mainly their *containment* relationships) are also less consistent with respect to the underlying ontology hierarchy. Considering a class *C* that has only a single subclass $D \sqsubseteq C$, a suboptimal placement of ${{\mathcal {B}}_{C}}$ would lead to a poor recall and consequently a poor F1 score for class *C*.

The performance for macro-aggregated metrics is significantly worse than the weighted versions. As discussed in Sect. 2, macro aggregation weights all classes equally, which puts a higher focus on very specific classes in ontology-based approaches, compared to weighted averaging. Our training dataset includes 854 classes, with 166 having only one subclass in the ontology.

In these cases, if the box model fails to infer the only existing subclass due to the imperfect placement of the child box, the F1 score for the respective class is considered 0 in the macro-averaging method, significantly affecting F1 results. Therefore, during training, the $w^{\text {norm}}$ weighting puts a higher emphasis on those very specific classes and therefore yields considerably better scores for the macro aggregated metrics for the subclass prediction. This, however, comes at the cost of worse performance in all other evaluation metrics, which is consistent with our analysis of the classification task.

**Table 3:** Derived superclasses and subclasses on training labels

			Weighted			Macro		
			Precision	Recall	F1	Precision	Recall	F1
Superclasses	${\varepsilon _{\text {sub}}}= 0.00$	$w^{\text {Cui}}$	**0.900**	**0.811**	**0.853**	**0.847**	**0.803**	**0.825**
		$w^{\text {norm}}$	0.858	0.633	0.728	0.786	0.631	0.700
	${\varepsilon _{\text {sub}}}= 0.03$	$w^{\text {Cui}}$	**0.894**	**0.849**	**0.871**	**0.838**	**0.858**	**0.848**
		$w^{\text {norm}}$	0.848	0.671	0.749	0.776	0.675	0.722
Subclasses	${\varepsilon _{\text {sub}}}= 0.00$	$w^{\text {Cui}}$	**0.825**	**0.811**	**0.818**	0.408	0.251	0.311
		$w^{\text {norm}}$	0.772	0.633	0.695	**0.604**	**0.391**	**0.475**
	${\varepsilon _{\text {sub}}}= 0.03$	$w^{\text {Cui}}$	**0.833**	**0.849**	**0.841**	0.541	0.383	0.449
		$w^{\text {norm}}$	0.770	0.671	0.717	**0.700**	**0.557**	**0.620**

### Disjoint and overlapping classes

Besides the subsumption relations, ontologies also specify other relations between classes. Two that are meaningful in the context of boxes are disjointness and overlaps. Classes are disjoint if they cannot share any members, e.g., *cyclic acid anhydride* and *acyclic acid anhydride*. Classes overlap if they can share members. For instance, a molecule can be both a *cyclic acid anhydride* and *carboxylic anhydride* (ChEBI knows 22 molecules which are subclasses of both, e.g., *glutaric anhydride*). While every pair of classes falls into one of these two categories, for most pairs, this is **unknown**. If there is no instance that belongs to both classes and no axiom that states that the classes have to be disjoint, we have no information about which category the class pair belongs to. For instance, there is no molecule in ChEBI that is both an *acid anhydrides* and a *selenium molecular entity* (even though such a molecule would be chemically feasible). Therefore, we consider disjointness and overlapping as separate properties of classes. In the following, we evaluate how well the box embeddings are able to reproduce these properties.

For many classes, ChEBI provides explicit disjointness axioms in a separate ontology module.^2^ We used these axioms to compute the deductive closure of disjointness between label classes. This means that for every axiom stating that *A* and *B* are disjoint, we also consider that *C* and *D* are disjoint if *C* is a subclass of *A* and *D* is a subclass of *B*. In total, 49,212 label pairs are explicitly disjoint. We consider these pairs as positive labels and all others as negative labels. Indeed, in ChEBI, neither the disjointness axioms nor the overlap of classes are completely represented. The latter means that not for every pair of overlapping classes, a common subclass is present.

**Table 4:** Recall, precision, and F1 for disjointness and overlap relations

		Disjointness								
		Micro			Weighted			Macro		
		Precision	Recall	F1	Precision	Recall	F1	Precision	Recall	F1
${\varepsilon _{\text {dis}}}= 0.00$	$w^{\text {Cui}}$	0.074	**0.992**	0.139	**0.469**	**0.992**	**0.637**	**0.074**	**0.989**	**0.138**
	$w^{\text {norm}}$	**0.075**	0.980	**0.139**	0.468	0.980	0.634	0.073	0.975	0.135
${\varepsilon _{\text {dis}}}= 0.03$	$w^{\text {Cui}}$	**0.074**	**0.994**	**0.137**	**0.469**	**0.994**	**0.637**	**0.076**	**0.991**	**0.142**
	$w^{\text {norm}}$	0.074	0.984	0.137	0.467	0.984	0.633	0.072	0.978	0.133

		Overlap								
		Micro			Weighted			Macro		
		Precision	Recall	F1	Precision	Recall	F1	Precision	Recall	F1
${\varepsilon _{\text {dis}}}= 0.00$	$w^{\text {Cui}}$	**0.975**	0.372	0.538	**0.981**	0.372	0.505	**0.963**	0.350	0.486
	$w^{\text {norm}}$	0.947	**0.400**	**0.562**	0.961	**0.400**	**0.531**	0.930	**0.375**	**0.502**
${\varepsilon _{\text {dis}}}= 0.03$	$w^{\text {Cui}}$	**0.987**	0.334	0.499	**0.991**	0.334	0.500	**0.977**	0.313	0.474
	$w^{\text {norm}}$	0.964	**0.353**	**0.517**	0.976	**0.353**	**0.519**	0.949	**0.329**	**0.489**

The results of this evaluation are shown in Table 4. Ideally, for the explicitly disjoint pairs, the corresponding boxes should not have any overlap. This is represented by the micro-recall. We see that, for the $w^{\text {Cui}}$ model, 99.2% of box pairs that are explicitly disjoint do not overlap at all and many of the overlapping pairs only overlap slightly. In absolute terms, this corresponds to 48,802 pairs. For the $w^{\text {norm}}$ model, this value is lower with 98.4% (48,252 pairs).

Although the precision scores are low, there is a strong justification for this outcome: disjointness axioms are not exhaustively expressed in the ChEBI ontology. Therefore, the low precision scores result from the fact that most classes with non-overlapping boxes are not explicitly defined as disjoint by the ChEBI ontology, although they should be. Still, it remains unclear whether this outcome is a limitation of the model or a result of missing disjointness axioms in the ontology. To approximate this, we need to examine the classes that are presumed not to be disjoint. We assume this because, for those class pairs, there is at least one molecule in the ontology that belongs to both classes. In total, 194,336 pairs share at least one molecule (26.6% of all pairs between classes with at least 100 members).

Here, the precision is significantly higher, ranging between 0.93 and 0.99 depending on the model and aggregation method. This means that most classes that were predicted to have an overlap are classes that also share molecules in the dataset. Only rarely, the models predict overlaps that are *″unnecessary″*, i.e., not required to classify the molecules in ChEBI correctly. Out of these non-required overlaps, most also do not contradict the disjointness axioms, as we have seen for the disjointness recall. Overall, the $w^{\text {Cui}}$ model has a lower tendency to produce unnecessary overlaps (2.5%) compared to the $w^{\text {norm}}$ model (5.3%). As can be expected, this tendency shrinks rapidly if the boxes are defined more tightly. The recall for overlaps shows the proportion of class pairs in ChEBI that have overlapping boxes. This number ranges between 0.3 and 0.4 (depending on the model and aggregation method), meaning that 30 to 40% of the classes that have shared molecules in ChEBI are represented by overlapping boxes, while the remaining class pairs have non-overlapping boxes.

One possible explanation for this behavior is that many class pairs only share a few molecules. The model then treats those molecules as outliers or simply does not receive a strong enough loss signal to change the box dimensions to incorporate these molecules.

Indeed, class pairs that are represented with non-overlapping boxes share significantly fewer members (on average, 12.7 / 13 members for $w^{\text {Cui}}$ and $w^{\text {norm}}$ models, excluding class pairs that do not share any members) than class pairs for which the boxes overlap (1205.7 / 1120.8 members). This confirms our hypothesis that class pairs that share only a few molecules are more likely not to be well-represented in the box models.

### Logical consistency

So far, we have only evaluated consistency on the level of classes, i.e., if the relations between boxes are in line with the class-relations in the ontology. There, we have seen that not all hierarchical or disjointness relations are represented perfectly by the boxes. For instance, in many cases, a small part of a subclass box is outside its superclass box (cf. Figure 8).

This raises a question for the practical application of the model: If we make predictions based on these boxes, how consistent will the results be? For instance, if a subclass box is not perfectly contained in a superclass box, there are four possibilities: The molecule can be predicted to be in both boxes, only in the superclass box or in none of the boxes, all of which would be consistent predictions, i.e., not in violation of the class relations. Which of these predictions is correct can be decided based on the labels (see Table 1). However, the fourth option, namely that the molecule is only in the subclass box and not in the superclass box, is a misclassification not due to the labels but due to the class relations. Therefore, we consider this prediction as inconsistent.

For instance, the $w^{\text {Cui}}$ box for *glycan* is mostly (but not fully) contained in the box of its superclass (*carbohydrates and carbohydrate derivatives*). Out of the 728 samples in our test set that the $w^{\text {Cui}}$ model has placed in the *glycan* box, 716 are also placed in the box for the superclass. The remaining 12 samples (e.g., CHEBI:147792) have an inconsistent prediction: they are classified as glycans, but not as carbohydrates.

**Table 5:** FNR for logical consistency of predictions with the subsumption and disjointness relations

		Micro	Superclasses		Subclasses	
			Weighted	Macro	Weighted	Macro
Subsumption	$w^{\text {Cui}}$	0.0398	0.0398	0.1080	0.0398	0.0305
	$w^{\text {norm}}$	**0.0012**	**0.0012**	**0.0102**	**0.0012**	0.0278
	Electra	0.0081	0.0081	0.0282	0.0081	**0.0258**
Disjointness	$w^{\text {Cui}}$	$8.67 \times 10^{-4}$	$8.67 \times 10^{-4}$	$5.14 \times 10^{-5}$	$8.67 \times 10^{-4}$	0.0037
	$w^{\text {norm}}$	$\mathbf {4.17 \times 10^{-7}}$	$\mathbf {4.29 \times 10^{-7}}$	$\mathbf {1.30 \times 10^{-8}}$	$\mathbf {4.77 \times 10^{-7}}$	$\mathbf {1.06 \times 10^{-8}}$
	Electra	$2.50 \times 10^{-6}$	$2.48 \times 10^{-6}$	$2.58 \times 10^{-5}$	$2.54 \times 10^{-6}$	$4.20 \times 10^{-5}$

Table 5 shows the proportion of inconsistent predictions for subclass-superclass pairs out of all predictions in which the subclass was predicted (false negative rate, FNR). The $w^{\text {norm}}$-model has a FNR with 0.0012, which is significantly lower than the FNR of the Electra model (0.0081), which outperforms the $w^{\text {Cui}}$-model (0.0398). For disjoint classes, we followed an analogous approach, for each datapoint we calculated the ratio between the pairs of positive predictions of classes that violated some disjointness axioms and the number of disjointness axioms, where one of the classes was predicted. Compared to the FNR on subsumptions, here, the FNR is several magnitudes lower (see Table 5). However, we observe again that the $w^{\text {norm}}$ model performs best with a FNR of $4.17 \times 10^{-7}$, followed by Electra ($2.50 \times 10^{-6}$) and $w^{\text {Cui}}$ ($8.76 \times 10^{-4}$).

### Extension with unseen classes

In order to simulate the inclusion of new classes into the existing hierarchy, we included classes that had at least 50 instances, thereby extending the set of labels, which was previously comprised of only classes with at least 100 instances. We then took all instances of each individual class and computed the bounding box of their embeddings. We then calculate the subsumption relation between new and existing boxes in the same way as when analyzing the subsumption relations of boxes in Sect. 3.1 and also evaluate them in the same way.

**Fig. 9:**
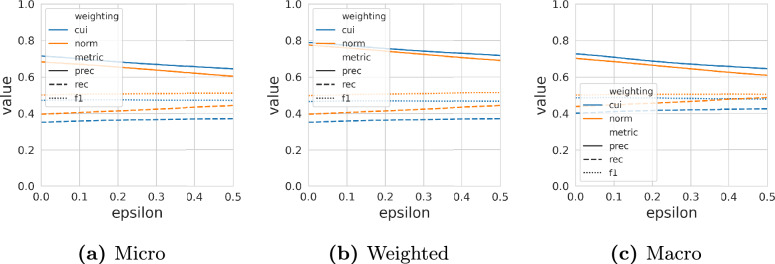
Metrics for zero-shot learning under different margin factors ${\varepsilon _{\text {sub}}}$

Figure 9 depicts the performance w.r.t precision, recall, and F1 for this evaluation for different values of ${\varepsilon _{\text {sub}}}$. It can be seen that the model based on the $w^{\text {norm}}$ weighting outperforms the model trained using the $w^{\text {Cui}}$ weighting when considering recall and F1. However, for the precision metric, the $w^{\text {Cui}}$ weighted model performs consistently better. The high precision scores of both models indicate their ability to derive highly accurate classes. However, the moderate recall scores suggest that the models may not be capable of identifying all possible subsumptions, reflecting the inherent complexity of the task. Distinct from all other evaluations, there is no large difference between micro and macro aggregation. All aggregations report an F1 score of around 0.5. This is likely due to the different distribution of classes. In all of the above analyses, there were some classes that had a large number of parents, children, or members. Classes considered in this analysis, however, reside mostly in the lower part of the ontology and have a roughly similar number of superclasses.

## Discussion

Our proposed model learns the geometry of boxes based on the structural annotations (SMILES strings) in the ChEBI ontology. For certain ChEBI classes, structural annotations are rare, hence, it is difficult to precisely learn their corresponding boxes in the embedding space. In other words, the different number of *per-class* annotations in ChEBI, combined with the hierarchical nature of the classes, resulted in a highly imbalanced training dataset. To address this problem, we used two different weighting strategies for the loss function of our model. Changing the weighting strategy makes it possible to have some control over the type of predictions the models produce. Assigning more weight to rare classes leads to more specific and potentially more interesting predictions from the perspective of chemists.

As indicated in earlier studies, the effectiveness of geometric embeddings is particularly evident in low dimensions but tends to diminish in higher dimensions [21]. We observed that the performance of our proposed box model tends to deteriorate in dimensions higher than 16. In our view, the increase in dimensionality leads to a more complex geometric space, which poses a challenge in accurately capturing the relationships between instances and classes. As a result, the computational complexity increases with higher dimensions, which can lead to optimization issues due to the curse of dimensionality. This issue is important when dealing with sparse data. For example, in recommendation engines or multi-label tagging systems, the focus is on excluding most of the items [19]. Likewise, in our model, due to the ontological nature of the training data, certain classes should have a high degree of overlap, whereas many classes are expected to have no overlap. As an example, classes that are deeply nested in the hierarchy and located near the leaf nodes often have less overlap. Therefore, as stated in [5], and illustrated in Fig. 3, the subsumption relations in ChEBI can be outlined as a diamond-shaped graph structure.

Lastly, we have shown that our system can be used to include new classes into an existing taxonomy. This feature is particularly useful because it addresses an important use case in ontology development. Our system may be used to build a new ontology from data points and then to classify new members in this ontology. Such classification systems have already been used in active ontology development. ClassyFire, a classification tool for chemicals, is actively used as part of ChEBI’s development process. Being a rule-based system does make it particularly hard to extend to new classes because new hand-written rules need to be derived. This rigidity then produces artifacts within the ontology itself. ChEBI’s branch of *peptide* (CHEBI:16670) shows, despite its importance in biochemical processes, a rather shallow subclass structure, in which most classes are represented as a direct subclass of *peptide* (CHEBI:16670). This structure has likely been caused by automated tools such as ClassyFire, which do not represent classes that are more specialized than *peptide* (CHEBI:16670). While newer systems such as Chebifier [11] address this issue by providing a machine-learning-based model, the inclusion of new classes still requires retraining the whole model. The zero-shot learning capabilities demonstrated by our model allow for a simpler approach. As we have shown, extensional box representations of classes can be derived from the embeddings of their members.

### Related work

Our contribution in this paper is three-fold: First, we created a system for the classification of chemical substances into classes from the ChEBI ontology. Second, the system allows insights into whether the model learned correct logical relations between labels. Third, our model allows dynamic bounding-box-based extensions with new labels that do not require retraining.

Previous work [5] also explored the applicability of traditional classifiers to the task of ontology extension. Some of these approaches (e.g. decision trees) also allow insights into learned decision rules. This analysis did however show that these approaches show a significantly worse performance than an LSTM-based model, which was later in-turn outperformed by a transformer-based model [6]. Notably, the transformer-based model achieved similar performance to the one proposed in this work, but did not provide the additional evaluation capabilities.

$\hbox {Box}^2$EL [10] extends the approach used for EL Embeddings to *n*-dimensional, axis-aligned boxes that extend this formalism to represent binary relations as pairs of boxes – one for each side of the relation. This allows this formalism to also capture role inclusions. $\hbox {Box}^2$EL puts the focus on the inclusion of an existing ontology into the learning process. More specifically, contrary to our approach, not only classes are embedded as boxes, but also individuals. These individual embeddings are model parameters that are part of the training procedure. It is therefore not obvious how this approach can be extended to embed and classify unseen entities. The approach proposed in this work includes a full input to the prediction pipeline that can process any molecular structure represented by a valid SMILES string.

In the context of Gene Ontology (GO, [22]), ontology-based knowledge has been integrated into the training step of deep learning methods in previous works. In DeepGO [23], the GO taxonomy is built directly into the architecture of the model. In the prediction step, it is used to modify the predictions of the model to ensure that all predictions are always consistent with the GO taxonomy. It should be emphasized that this “repair process“ has no influence on the actual learning process. The model itself is not encouraged to provide more consistent answers, as the ontology layer does not return a gradient to the trainable layer during these repairs.

Geometric representations of ontology classes are particularly related to our work. EL Embeddings [9] is a method for representing ontology classes in the ${{\mathcal {E}}}{{\mathcal {L}}}^{++}$ ontology language using *n*-dimensional balls and relations as linear translations. A loss function is defined using the axioms of the ontology, utilizing the fact that ${{\mathcal {E}}}{{\mathcal {L}}}^{++}$ ontologies can be represented using 5 normal forms. By optimizing this loss function, it is then possible to learn a geometric representation of the classes that represent the logical axioms of the ontology.

These loss definitions have also been employed in DeepGOZero [24], where the ball embeddings are co-trained with additional embeddings for proteins in order to predict protein functions. Notably, it is possible to extend this approach to allow for zero-shot predictions on unseen classes. A geometric representation of classes as *n*-dimensional balls does not preserve certain properties such as closedness under intersection: Given two *n*-dimensional balls, their intersection is not an *n*-dimensional ball, unless one contains the other. A box-based representation, however, preserves this property. Consequently, certain axioms that cannot be represented using balls can be represented using boxes, e.g., $A \equiv B \sqcap C$. Embeddings as axis-aligned boxes overcome this downside because their intersection yields, again, an axis-aligned box. As we have discussed in Sect. 3.4, our approach can also be used to represent such axioms and, further, allow the discovery of new axioms of this form.

$\hbox {Box}^2$EL [10] and DeepGOZero [24] also include the ontology axioms directly into the training process. Our methodology does not intentionally include the explicit taxonomical information in the training process in order to use it as an evaluation criterion instead.

Primarily, in tasks related to the prediction of class labels with a hierarchical nature, it is crucial for the model to respect this structure [20]. Vector embeddings are widely used for capturing semantic similarities, but these methods, in their basic form, often struggle to model complex hierarchical and asymmetrical relationships [21, 25–29]. The limitation of vectors in effectively modeling complex interdependencies between labels is also emphasized in [20]. Earlier works on multilabel prediction often rely on dot products or cosine similarity to evaluate their relatedness [30–32]. To model asymmetrical relationships such as class subsumption, additional mechanisms are often required. In comparison, box embedding offers a more expressive and suitable approach for representing hierarchies. Basically, embedding methods based on geometric regions excel at capturing directional edges via the notion of geometric containment [21]. It has also been shown that box embeddings have proven effective not only for representing tree-like structures but also for modeling graphs with nodes having multiple parents and capturing intersections between different classes. [20, 29, 33].

Whilst our proposed method focuses on the application on chemical class prediction, Box embedding methods have been explored with applications in knowledge bases. The method used in [34] represents label relationships as conditional probabilities, and [25] combines geometric and probabilistic views to represent boxes. The approach introduced in [35] employs a hyperbolic Poincaré ball model and represents labels as Poincaré hyperplanes. From another perspective, [36] views knowledge bases as an organization of facts through relationships between entities and employs a *spatio-translational* approach to embed entities as points while representing relationships as hyper-rectangles. The proposed method in [37] learns a joint hierarchy from *is_a* and *has_part* relations and introduces a transformation technique to adjust box representations across hierarchies by translating and expanding them. While in principle similar to our proposed model, these approaches differ in the kind of data that is used to derive these geometric representations. Their embeddings are derived from natural language embedding WordNet, which covers a variety of natural language fragments. It does not however cover chemical structures. These methods therefore, cannot be easily applied to chemical compounds.

Our proposed approach differs from previous methods in several aspects. While methods introduced in [34, 38, 39] have a *probabilistic* perspective on boxes, and [25] combines geometric and probabilistic views to represent boxes, we take a geometric approach and represent boxes as *rigid* products intervals in an n-dimensional space. The probabilistic view addresses the problem of lack of local identifiability, which means that different box positions and sizes can lead to the same loss, potentially leading to flat regions in the loss landscape, with near-zero gradients. In order to address local identifiability in a lightweight way, during training, we used *soft* memberships with a sigmoid function to determine *point-to-box* associations, allowing direct utilization of the binary cross-entropy loss. This membership function is explained in Sect. 2. The method introduced in [40] incorporates label associations as constraints into the score function during training. Our lightweight approach of softened rigid boxes (using a margin-based loss) avoids the complexity of probabilistic volume calculations and also provides a more intuitive geometric interpretation of the ChEBI class hierarchy — after all, soft box membership and containment are conceptually easier than a probabilistic approach.

### Discovering potential axioms

A key property of box embeddings is that the intersection of two boxes is itself a box. This geometric characteristic allows us to leverage the Jaccard index [41], also known as Intersection over Union (IoU) measure, to identify existing boxes that closely match such intersections, enabling the discovery of candidate equivalence axioms of the form $X \equiv Y \sqcap Z$. The IoU is a well-known metric used to quantify the degree of overlap between two sets. For boxes, it is computed as the ratio of the intersection to the union area. Using box embeddings for chemical classes, IoU determines the degree of similarity between two chemical classes by comparing the spatial overlap of their box representations. A higher IoU means the classes are more closely related.

In some initial experiments, we explored the potential of the IoU for discovering new axioms for ChEBI. Our algorithm operates on the learned box embeddings of chemical classes from the ChEBI ontology. It begins by identifying all pairs of overlapping boxes that are not hierarchically related via a subclass relationship. For each such pair, it checks for a third box that closely resembles their intersection, while assessing how closely the intersection of two overlapping boxes aligns with another existing box in the vector space. By utilizing the IoU metric, we considered only cases where the value exceeds 0.99, ensuring that only highly similar matches are selected. Such cases correspond to axioms expressed as $X \equiv Y \sqcap Z$ that have been learned from the data without an explicit signal during training (see Sect. 2.3). Table 6 presents the results of our algorithm using $w^{\text {Cui}}$, highlighting potential equivalence axioms, which are absent in the ChEBI ontology. These initial results indicate that our proposed box embedding approach may be used beyond learning a taxonomic hierarchy, but as a method for discovering more complex axioms. In the future, we are planning to continue this line of research.

**Table 6:** Potential axioms of type $X \equiv Y \sqcap Z$ from the model trained with $w^{\text {Cui}}$ weights

Equivalent class		Intersecting classes
organic heterobicyclic compound	$\equiv$	organic heterocyclic compound $\sqcap$ heterobicyclic compound
organic heteromonocyclic compound	$\equiv$	heteroorganic entity $\sqcap$ heteromonocyclic compound
heteromonocyclic compound	$\equiv$	heteroorganic entity $\sqcap$ monocyclic compound
monocyclic compound	$\equiv$	heteroorganic entity $\sqcap$ heteromonocyclic compound
organic heteropolycyclic compound	$\equiv$	heteroorganic entity $\sqcap$ heteropolycyclic compound
carbocyclic compound	$\equiv$	homocyclic compound $\sqcap$ organic molecular entity
carbopolycyclic compound	$\equiv$	organic cyclic compound $\sqcap$ homopolycyclic compound
organic fundamental parent	$\equiv$	organic hydride $\sqcap$ organic molecular entity

## Conclusion

In this paper, we presented two models ($w^{\text {norm}}$-model, $w^{\text {Cui}}$-model) that are able to automatically classify chemical structures with terms from ChEBI. These models are identical except for the weighting schemes in their loss function. In contrast to previous works, we used an approach that enabled us to inspect whether the representation of ChEBI’s class hierarchy within the trained model matches ChEBI. This is achieved by co-training the embedding of chemical structures in the embedding space and the representation of classes as boxes in the embedding space. While this architecture enables more transparency, it adds constraints to the possible representation of classes in the embedding space. Nevertheless, the trained models perform with respect to the classification of chemicals on par with the baseline and previous work.

While the classification performance of the models is on par, they differ with respect to the logical consistency with the axioms of ChEBI. The classifications of chemical entities by the $w^{\text {norm}}$-model are significantly more logically consistent with ChEBI axioms than the baseline Electra model, which itself significantly outperforms the $w^{\text {Cui}}$-model.

One major benefit of our approach is the ability to compare the learned ontology with the actual ontology since classes are represented as boxes in the embedding space. As presented in Sect. 3, the models learn the subsumption hierarchy quite successfully. Precision, recall, and F1-measure of the micro aggregation of the subsumption hierarchy as well as the weighted and macro aggregation based on superclasses are, in the 0.8$-$0.9 range (for the $w^{\text {Cui}}$-model). However, the model performs worse for classes with few subclass relationships. These are classes that are lower in the taxonomic hierarchy and, thus, typically, have comparatively few instances.

Disjointness between classes in ChEBI is more difficult to learn. One reason is that the model has no possibility to distinguish whether two classes *A*, *B* are disjoint or whether ChEBI just accidentally contains no example of a chemical structure that instantiates both *A* and *B*. In these cases, the model represents the classes as non-overlapping boxes. Thus, the precision of predicting disjointness axioms is low. However, if two classes are disjoint, the model learns it reliably (recall > 0.99). Consequently, the classification of chemical structures by the model is – with only a few exceptions – logically consistent with the disjointness axioms in ChEBI.

Another advantage of our approach is that it provides the possibility of extending an ontology with classes that are not part of the training set (zero-shot learning). Our training dataset included 854 labels of classes that have at least 100 molecules as members of ChEBI. One reason to limit our approach to these classes is that Transformer models need a certain number of examples for learning. The semantics of classes that were not included in the training dataset, but that contain known members within this dataset, can be approximated by considering the bounding box of the embedding of their members in the embedding space. To evaluate this approach, we applied it to 9805 ChEBI classes, which have between 2 and 99 members. Thus, we extended the taxonomic hierarchy more than tenfold and with classes that are difficult to learn because of a lack of data. F1-measures of roughly 0.5 for this difficult task illustrate that the approach shows promise, and offers significant potential for improvement.

## Future work

As mentioned in Sect. 4.2, we plan to investigate the potential of our methodology for discovering new axioms more systematically. Further, we plan to examine different geometric representations of classes in the latent space, e.g., cones, spheres, and polytopes [42–45]. We will also explore different methods to increase the semantic significance of the latent space, e.g., by adding a similarity-based pull to the gradient. Another topic is learning more complex rules than subsumption, disjointness, and overlap, cf. rule learning [46, 47].

We showed how box embeddings, when applied to ontologies, can map subsumption relations and class assertions into relations among boxes and relations among points and boxes, respectively. In the process of learning the memberships of molecules to their respective classes, the proposed method uncovers the associations between classes themselves in the absence of any explicit training signal about the label relationships. Representing SMILES strings as points and labels as hyper-rectangles in a shared latent space allows for imposing implicit constraints on the embedded molecules, as they need to be placed inside the boxes representing the classes to which the molecules belong. The utilization of *point-in-box* constraints introduces a geometric bias into the learned representations of molecules. This geometric bias encourages similar molecules to be clustered in the latent space, giving the embedded molecules more meaningful geometric proximity in relation to their chemical properties. Ultimately, it promotes learning conceptual spaces in the chemistry domain with interpretable embedding dimensions. Future work will extend our present work using results about conceptual spaces [48].

## Data Availability

All implementations, including training and evaluation scripts, are publicly accessible from the GitHub repository: https://github.com/adelmemariani/python-chebai/tree/Box4Chemi. Trained models, associated datasets, and evaluation results are publicly accessible at: https://doi.org/10.5281/zenodo.15286967.

## Footnotes

1 The same approach may be used to include any chemical class that is not already contained in ChEBI (e.g., from another ontology). The only requirement is a mapping from that class to its members that are in ChEBI or a different collection of SMILES strings contained in this class.

2 See https://ftp.ebi.ac.uk/pub/databases/chebi/ontology/chebi-disjoints.owl
